# Effectiveness of exercise intervention on physical and health outcomes in patients admitted to an acute medical ward: A systematic review and meta-analysis

**DOI:** 10.1177/02692155241240637

**Published:** 2024-03-27

**Authors:** Jane L McCaig, Brett A Gordon, Carolyn J Taylor

**Affiliations:** 1 La Trobe Rural Health School, 2080 La Trobe University, Bendigo, Australia; 2 Holsworth Research Initiative, 2080 La Trobe University, Bendigo, Australia

**Keywords:** Exercise intervention, acute medical ward, hospital-acquired functional decline, hospital associated disability

## Abstract

**Objective:**

To evaluate the effectiveness of inpatient medical ward exercise on physical and health outcomes in adults compared with usual care.

**Data sources:**

Medline, CINAHL and EMBASE were searched from inception to 20 April 2023.

**Review methods:**

Randomised-controlled trials in English that reported physical and health outcomes of adults who received an exercise intervention on an acute medical ward were included. Two reviewers independently extracted data. Methodological quality was assessed using the PEDro and TESTEX scales. The GRADE rating assessed the quality of evidence to evaluate the certainty of effect. Meta-analyses were performed where possible.

**Results:**

Thirteen studies were included, with 1273 unique participants (mean [SD] age, 75.5 [11] years), which compared exercise intervention with usual care. Low quality evidence demonstrated a significant improvement in aerobic capacity ([MD], 1.39 m [95% CI, 0.23, 2.55], p = 0.02) and maximum isometric strength ([MD], 2.3 kg [95% CI, 2.2, 2.4], p < 0.001) for the exercise intervention compared with usual care. Low quality evidence demonstrated no difference for in-hospital falls count ([OR], 1.93 [95% CI, 0.61, 6.12] p = 0.27) or mortality ([OR], 0.77 [95% CI, 0.48, 1.23], p = 0.27). Moderate quality evidence demonstrated no difference for length of stay ([MD], −0.10 days [95% CI, −0.31, 0.11] p = 0.36).

**Conclusion:**

Exercise prescribed during an acute medical ward stay improves aerobic capacity and maximum isometric strength but may not reduce length of stay, in-hospital falls or mortality.

## Introduction

A hospital stay in an adult population can lead to negative health consequences, even after the reason for acute hospitalisation has been treated and resolved.^1^ An acute hospital stay is associated with large amounts of sedentary time, with most patients undertaking bed rest and low amounts of physical activity for the stay duration.^2^ Up to 75% of hospitalised older adults do not walk at all during their hospital stay,^3^ potentially contributing to poor health outcomes. Hospital-acquired functional decline is the term given for the decrease in physical ability following a hospital stay^4^ and affects up to one-third of hospitalised older adults.^1^ Hospital-acquired functional decline has been associated with increased disability risk, which can lead to increased resource use and poorer health outcomes.^5^ Strategies that lead to a reduced rate of hospital-acquired functional decline could improve individual health outcomes and reduce the burden on health service delivery.

Exercise prescription on the acute inpatient medical ward can prevent decline or at least maintain physical outcomes in the adult population.^6–10^ Previous reviews have examined physical function (the combination of physical outcomes) as an umbrella term, not allowing for delineation of the effects of an on-ward exercise intervention on aerobic capacity, muscle strength and balance. Although understanding the effect on global physical function can be important, it does not help to stratify or manage risk. Identifying the effects of on-ward exercise on specific physical outcomes might also allow for a targeted exercise prescription to optimise physical ability for acute medical ward inpatients and ensure resources are utilised in the most effective manner.

The purpose of this systematic review and meta-analyses of randomised-controlled trials was to evaluate the effectiveness of exercise intervention on the acute inpatient medical ward on physical and health outcomes in adults compared with usual care. The primary aims were to evaluate the effect of a ward-based exercise intervention on the physical outcomes of muscle strength, aerobic capacity and balance; and secondary aims were to evaluate any effects on the health outcomes of length of stay, readmissions, falls count and mortality.

## Methods

This review was prospectively registered with PROSPERO (ID: 42021254505) and was reported in accordance with the Preferred Reporting Items for Systematic Reviews and Meta-Analyses (PRISMA) guidelines.^11^

Studies were included in the review if they met the following criteria: i) were original research randomised-controlled trials (RCTs); ii) were conducted in adults aged 18+ years admitted to a hospital acute medical ward; iii) had a group that received exercise prescribed on the acute medical ward and the exercise prescribed comprised of any combination of aerobic exercise, resistance exercise and balance exercise; iv) had a control group that received usual care or no exposure to exercise intervention.

Studies were excluded if: i) they were observational or cohort studies, literature reviews, systematic reviews or scoping reviews; ii) the exercise intervention was conducted on inpatient rehabilitation wards, surgical wards or outpatient clinics; iii) the participant population included acute stroke participants (as likely to be discharged to a rehabilitation ward); iv) they were published in a language other than English; v) they did not include any outcome measure for the prescribed exercise or any health outcome.

For this review, physical outcomes were defined as aerobic capacity, muscle strength and balance. Health outcomes were length of stay, falls count, readmissions, and mortality, as defined by the Australian Institute of Health and Welfare.^12^ These health outcomes were selected as they are routinely recorded during a hospital admission and are common research outcomes.^12^

Potential studies were identified by searching the Medline, EMBASE and CINAHL electronic databases from the earliest available date until 20 April 2023. The following search strategy was adapted to suit the individual database: (inpatient* OR “medical ward” OR hospital OR “acute medical” OR “hospital-acquired functional decline” OR “hospital-associated disability”) AND (exercise OR physiotherapy OR “physical thera*” OR “exercise physiology” OR nurse OR nurses) AND (“physical function” OR “physical capacity” OR “grip strength” OR strength* OR walk* OR “sit to stand”) AND (adult (MeSH) OR “middle age” OR adult 80+ OR adult^kw^). The full search strategy is listed in Supplemental Appendix 1.

The systematic search was performed by one author, and the citations were exported to Endnote^TM^, Version 20 (Thomson Reuters, New York, USA) and duplicates removed. The remaining citations were transferred to the Covidence^TM^ systematic review software (Veritas Health Innovation, Melbourne, Australia) and further screened for duplicates. Once all duplicates were removed, the retrieved citations were screened by title and abstract independently by two authors according to the pre-determined exclusion criteria. Disagreements were discussed and resolved between authors performing the initial screen. Full texts of remaining articles were reviewed and assessed independently by two authors. Any disputes were referred to the third author, and following discussion and consensus, a decision made.

Two authors independently extracted the following data from each study: number of participants within each group; participant characteristics (age, sex and medical diagnosis); exercise intervention characteristics (frequency, intensity, duration, time and type of exercise); physical outcome measures and health outcome measures.

Methodological quality was evaluated using the Physiotherapy Evidence Database (PEDro) scale^13^ with scores downloaded from the PEDro website,^14^ which is based on the Delphi list. Two authors independently assessed the methodological quality of the included studies using the Tool for the Assessment of Study Quality and Reporting in Exercise (TESTEX) scale, which provides a comprehensive review of exercise training trials.^15^ The TESTEX scale was used in conjunction with the PEDro scale as the TESTEX scale takes into consideration elements that are difficult to control for in exercise trials, such as participant blinding.^16^ Disagreements were resolved via a third author through discussion and consensus. The GRADE (Grading of Recommendations, Assessment, Development and Evaluations) scale was used to assess the quality of evidence.^16^

Statistical analyses were conducted using the Review Manager 5.4.1^TM^ software. Meta-analyses were performed when two or more studies presented data that compared the effectiveness of exercise with the usual care. Where studies reported continuous data for the same measure of assessment, weighted mean differences with 95% confidence intervals were computed with a fixed effects model to estimate the mean effect for each outcome. Where studies reported continuous data for different measures of assessment and reported post-test outcomes, standardised mean differences with a random effects model was used, with 95% confidence intervals. For studies with continuous data that reported different measures of assessment, expressed as a change from baseline scores, a weighted mean difference with a random effects model was used. For studies with dichotomous data that used the same measure of assessment, a fixed odds ratio with 95% confidence intervals was used. Where studies reported outcomes as median and interquartile ranges, conversion to mean and standard deviation were completed to allow the meta-analysis to be performed.^17,18^ The threshold for statistical significance was set at p < 0.05. Heterogeneity of studies was assessed using I^2^ statistic, with a score of <40% considered low, 40–59% moderate, 60–85% substantial and 86–100% considerable.^19^

## Results

**Figure 1.**
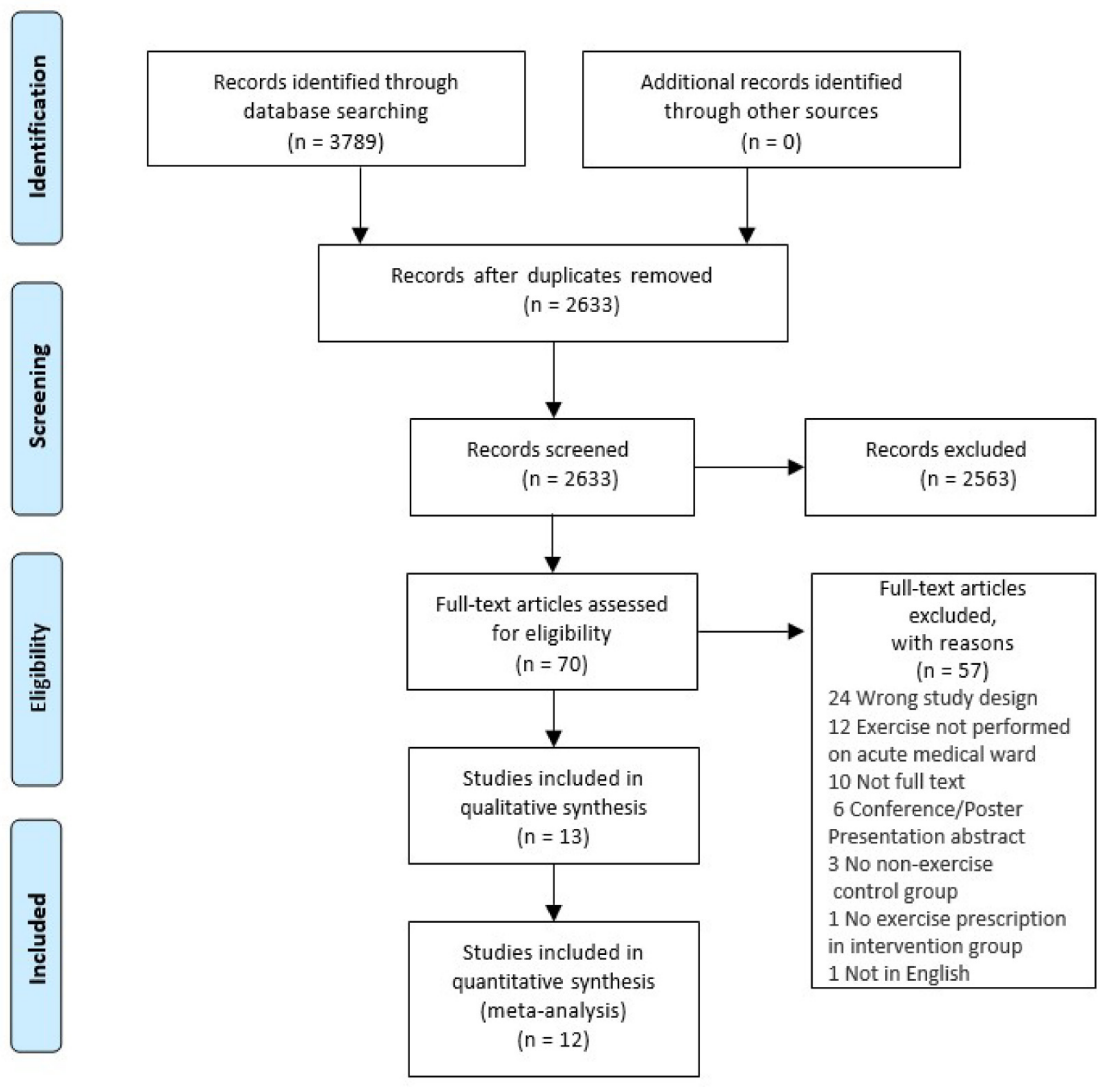
PRIMSA flow chart.

Thirteen studies were included in the systematic review^9,20–31^ with a total of 1273 participants (Figure 1). As one study^31^ was a secondary analysis of an original RCT,^9^ only data from the original RCT were included in the meta-analyses. The studies included between 16 and 370 participants, with an age range of 51 to 88 years, and all studies included male and female participants (Table 1). Seven studies included participants admitted for multiple medical reasons, including pulmonary, cardiovascular and gastrointestinal admission codes,^9,21,22^^,^^25–27,29^ three studies included participants only with respiratory conditions,^23,24,30^ one study included participants only with cardiovascular conditions^20^ and one study included participants only with type 2 diabetes.^28^ The characteristics of the studies are summarised in Table 1.

**Table 1.** Summary of study characteristics.

Study	Participant details	Demographics (female %) mean age	Intervention	Outcome measures	Main results
Ahmad et al. (2022)^20^	Medical ward, cardiovascular	Exp = 66 (53%) 78 years Con = 69 (55%) 80 years	ID = until discharge T = walking, resistance, balance F = 1× day D = not reported	*Physical*: FTSST; gait speed, semi-tandem stance, tandem stance *Health*: LOS	No significant differences between groups for health outcomes No reported differences in physical outcomes
Braun et al. (2019)^21^	Medical ward, geriatrics	Exp = 17 (72%) 83 years Con = 18 (76%) 79 years	ID = until discharge or maximum of three weeks I = not reported T = resistance, balance, walking exercises F = 4–5× week D = 20–25 minutes per session I = BORG 12–14	*Physical*: TUG; 6MWT *Health*: LOS	No differences between groups for physical or health outcomes
Hamilton et al. (2019)^22^	Medical ward	Exp = 50 (54%) 76 years Con = 52 (69%) 77 years	ID = until discharge T = walking, mobility F = 3x day D = not reported I = not reported	*Health*: LOS, falls, re-admissions	No differences between groups for health outcomes
Jose and Dal Corso (2016)^23^	Inpatient admitted with community-acquired pneumonia	Exp = 32 (47%) 51 years Con = 17 (41%) 59 years	ID = 8 days T = walking, resistance, stretching F = 1× day D = 45 minutes per session I = 70% max strength; 70% walk speed; 4–6 BORG	*Physical*: ISWT, max isometric strength (biceps brachii, deltoids, quadriceps, hamstrings)	Significant differences between groups for all physical outcomes
Kirsten et al. (1998)^24^	Acute medical ward, severe COPD	Exp = 15 (20%) 62 years Con = 14 (0%) 66 years	ID = 10 days T = walking F = 6× day D = not reported I = not reported	*Physical*: 6MWT	Significant differences between groups for physical outcome
Martinez-Velilla et al. (2019)^9^	Acute medical ward, geriatrics	Exp = 185 (54%) 88 years Con = 185 (59%) 87 years	ID = 5–7 days T = walking, resistance, balance F = 2× day D = 20 minutes per session I = 60% 1RM	*Physical*: hand grip strength *Health*: LOS, discharge destination, readmissions, falls, mortality	Significant differences between groups in physical outcomes No significant differences between groups for health outcomes
McCullagh et al. (2020)^25^	Acute medical ward, geriatrics	Exp = 95 (64%) 80 years Con = 95 (41%) 82 years	ID = until discharge T = walking, resistance, balance F = 2× day D = 20–40 minutes per session I = not recorded	*Health*: Falls, mortality, readmission, LOS	Significant differences between groups for readmission and mortality health outcomes No significant differences between groups for falls or LOS health outcomes
McGowan et al. (2018)^26^	Acute medical ward, geriatrics	Exp = 24 (67%) 87 years Con = 24 (54%) 83 years	ID = 7 days or until discharge if LOS < 7 days T = chair pedals F = 1× day D = 5 minutes per session I = not reported	*Physical*: Maximum isometric strength (quadriceps, hamstrings) *Health:* LOS	No significant differences between groups for physical or health outcomes
Ortiz-Alonso et al. (2020)^27^	Medical ward, geriatrics	Exp = 143 (60%) 88 years Con = 125 (54%) 88 years	ID = until discharge T = walking, LL strength F = 1-3× day D = 20 minutes per session I = self-selected and not recorded	*Health:* Falls, LOS, mortality	No significant differences between groups for health outcomes
Ozdirenc et al. (2004)^28^	Acute medical ward; type 2 diabetes	Exp = 23 (22%) 62 years Con = 21 (24%) 60 years	ID = until discharge T = walking, resistance, balance F = 2× day D = 20-45 minutes per session I = 13–14 BORG	*Physical*: 6MWT	Significant differences between groups for physical outcomes
Saez de Asteasu et al. (2019)^29^	Acute medical ward, geriatrics	Exp = 65 (49%) 88 years Con = 65 (49%) 86 years	ID = 5–7 days T = walking, resistance, balance F = 2× day D = 20 minutes per session I = 30-60% 1RM	*Physical*: FTSST, 1RM leg press *Health:* LOS	Significant differences between groups for physical outcomes No significant difference between groups for health outcomes
Salisbury et al. (2010)^30^	Acute medical ward post mechanical ventilation	Exp = 8 (38%) 67 years Con = 8 (25%) 58 years	ID = until discharge T = walking, resistance, balance F = not reported D = not reported I = not reported	*Health*: LOS, mortality	Significant difference between groups for LOS health outcome No significant differences between groups for mortality health outcome

Exp: experimental group; Con: control group; COPD: chronic obstructive pulmonary disease; ID: intervention duration; T: exercise type; F: exercise frequency; D: exercise duration; I: exercise intensity; TUG: timed up-and-go test; 6MWT: six-minute walk test; LOS: length of stay; LL: lower limb; ISWT: incremental shuttle walk test; FTSST: five times sit-to-stand test; BORG: rating of perceived exertion; 1RM: one-repetition maximum.

The quality of the included studies was fair to high on both the PEDro and TESTEX scales (Tables 2 and 3). Eight studies were deemed to be of fair quality^9,22–24,26–28,30^ and four of high quality.^21,22,25,29^ Studies were downgraded on the PEDro scale due to a lack of blinding of both therapists, assessors and participants. Studies were downgraded to fair quality on the TESTEX scale due to lack of reporting of exercise parameters, adaptation of relative exercise intensity and lack of monitoring of activity in the control group. The certainty of the evidence varied between low and moderate for the study outcomes (Table 4).

**Table 2.** Quality of included studies: PEDro scale.

Author (year)	1	2	3	4	5	6	7	8	9	10	11	Total
Ahmad et al. (2022)	1	1	0	1	0	0	1	1	1	1	1	8/10
Braun et al. (2019)	1	1	1	1	0	0	1	0	1	1	1	8/10
Hamilton et al. (2019)	1	1	0	0	0	0	0	0	1	1	1	4/10
Jose and Dal Corso (2016)	1	1	1	1	0	0	0	0	1	1	1	6/10
Kirsten et al. (1998)	1	1	0	1	0	0	0	1	0	1	1	5/10
Martinez-Velilla et al. (2019)	1	1	0	1	0	0	1	0	1	1	1	6/10
McCullagh et al. (2020)	1	1	1	1	0	0	1	1	1	1	1	8/10
McGowan et al. (2018)	1	1	1	1	0	0	0	1	0	1	1	6/10
Ortiz-Alonso et al. (2020)	1	1	0	1	0	0	0	1	0	1	1	5/10
Ozdirenc et al. (2004)	1	1	0	1	0	0	0	1	0	1	1	6/10
Saez de Asteasu et al. (2019)	1	1	1	1	0	0	1	1	1	1	1	8/10
Salisbury et al. (2010)	1	1	1	0	0	0	1	0	0	1	1	5/10

*Note.* Column numbers refer to the following criteria on the PEDro scale: 1: eligibility criteria; 2: random allocation; 3: allocation concealment; 4: similar baseline; 5: blind subjects; 6: blind therapists; 7: blind assessors; 8: 85% outcomes; 9: intention to treat analysis; 10: between groups comparisons; 11: point measures and variability. Total score out of 10 as criteria 1 is not scored.

**Table 3.** Quality of included studies: TESTEX scale.

Author (year)	1	2	3	4	5	6	7	8	9	10	11	12	Total
Ahmad et al. (2022)	1	1	0	1	0	3	1	2	1	0	0	0	10/15
Braun et al. (2019)	1	1	1	1	1	0	1	1	1	0	1	1	10/15
Hamilton et al. (2019)	1	1	0	0	0	0	1	2	1	1	0	0	7/15
Jose and Dal Corso (2016)	1	1	1	1	0	0	1	2	1	0	1	0	9/15
Kirsten et al. (1998)	1	1	0	1	0	1	0	2	1	0	0	0	7/15
Martinez-Velilla et al. (2019)	1	1	0	1	1	0	1	2	1	0	0	0	8/15
McCullagh et al. (2020)	1	1	1	1	1	0	1	2	1	1	0	0	10/15
McGowan et al. (2018)	1	1	1	1	0	2	0	1	1	0	0	0	8/15
Ortiz-Alonso et al. (2020)	1	1	0	0	0	3	0	2	1	0	0	0	8/15
Ozdirenc et al. (2004)	1	1	0	1	0	2	0	2	1	0	0	0	8/15
Saez de Asteasu et al. (2019)	1	1	1	1	1	3	1	2	1	0	1	0	13/15
Salisbury et al. (2010)	1	1	1	0	1	0	0	2	1	0	0	0	7/15

*Note.* Column numbers refer to criteria on TESTEX scale: 1: eligibility criteria; 2: random allocation; 3: allocation concealment; 4: similar baseline; 5: blind assessor; 6: 85% outcome measures (three points available for adherence >85%, adverse event reporting, attendance reporting); 7: intention to treat analysis; 8: between group comparison (two points available for primary outcomes and secondary outcomes); 9: point measures and variability; 10: control group activity monitoring; 11: relative intensity constant; 12: exercise volume and expenditure.

**Table 4.** GRADE assessment of evidence.

Outcome	No of studies	Study design	Risk of bias	Inconsistency	Indirectness	Imprecision	RATING
Aerobic Capacity	4	RCT	Not serious	Very serious	Not serious	Serious	Low
MIS	2	RCT	Not serious	Very serious	Not serious	Serious	Low
LOS	8	RCT	Not serious	Serious	Not serious	Not serious	Moderate
Falls	4	RCT	Not serious	Very serious	Not serious	Serious	Low
Mortality	4	RCT	Serious	Not serious	Not serious	Serious	Low

*Note.* 6MWT: six-minute walk test; MIS: maximum isometric strength; LOS: length of stay; RCT: randomised-controlled trial.

Exercise intervention was described in 12 studies. Seven studies included walking, strength-based and balance in the exercise protocol.^9,20,21,25,28–30^ Two studies included both walking and strength-based exercise ;^23,27^ walking was the single exercise intervention for two studies,^22,24^ and a chair-based pedal protocol was the only intervention for another.^26^

The duration of each exercise intervention session was reported in all but three studies.^20,22,30^ One study included an exercise duration of five minutes^26^ and all other studies reported exercise duration between 20 and 50 minutes.^9,21,23–25,27–29,31^ The frequency of exercise sessions was reported in all studies except one,^30^ with exercise completed four to five times per week,^21^ once per day,^20,23,26^ twice per day,^9,25,28,29^ one to three times per day,^27^ three times per day,^22^ or up to six times per day.^24^

Exercise intensity was self-selected by participants (but not measured or reported) in four studies^24–27^ and prescribed but not reported in three.^20,22,30^ The BORG scale^32^ was used to prescribe intensity in three studies^21,23,28^ of either six out of 10 or 12–14 out of 20. Walking speed, as a measure of exercise intensity, was used for exercise prescription in one study.^23^ Strength training intensity was prescribed at 30–60% one repetition maximum in two studies^9,29^ and at 70% of maximum strength in one study.^23^ The exercise interventions were supervised by trained staff (qualified allied health professionals or research assistants) in all studies except one, where the participants were unsupervised.^26^

### Physical outcomes

**Figure 2.**
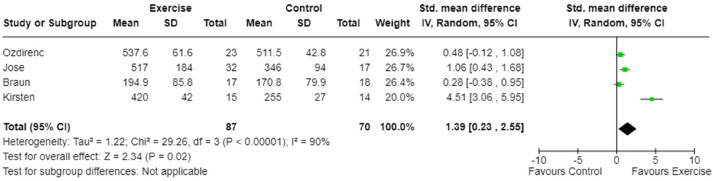
Meta-analysis of the effect of exercise intervention on aerobic capacity (walk distance).

Four studies assessed aerobic capacity via walking distance using the six-minute walk test^21,24,28^ or the intermittent shuttle walk test^23^ and enabled a meta-analysis. Meta-analysis revealed a significant improvement in aerobic capacity for exercise intervention compared with usual care interventions (p = 0.02, Figure 2); however, the certainty of evidence was low (Table 4), and heterogeneity was considerable (I^2 ^= 90%, Figure 2).

**Figure 3.**
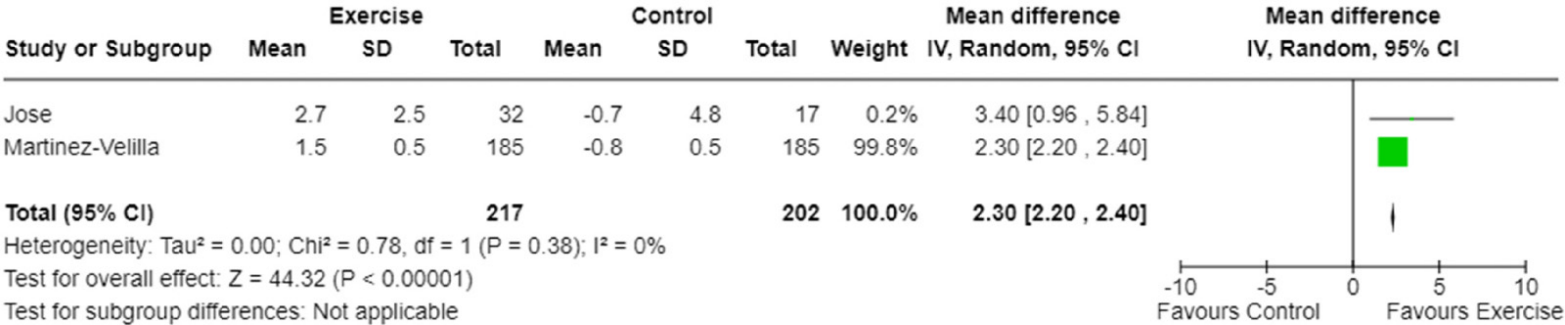
Meta-analysis of the effect of exercise intervention on maximum isometric strength.

Three studies measured maximum isometric strength.^9,23,26^ One study measured quadriceps, hamstrings, biceps brachii and deltoid muscle strength,^23^ one study measured quadriceps and hamstrings strength only,^26^ and one study assessed hand grip strength.^9^ One study was excluded from the meta-analysis for maximum isometric strength as appropriate data was not published and not provided after contact with the author.^26^ Therefore, two studies were included in the meta-analysis^9,23^ and assessed upper limb maximum isometric strength via grip strength and biceps brachii isometric strength. A significant effect for maximum isometric strength favouring exercise intervention was demonstrated (p < 0.001, Figure 3). The certainty of evidence was low (Table 4) with low heterogeneity (I^2^ = 0%, Figure 3). A single study measured dynamic strength using a one repetition maximum of a bilateral leg press.^29^ The study demonstrated a significant difference between groups for lower limb dynamic strength.

Balance was measured in one study using the timed up-and-go test.^21^The timed up-and-go is a measure of mobility and dynamic balance. It is validated to assess falls risk in older adults and easily performed in a clinical setting.^33^ A minimal clinical important difference range of 0.9–3.5 seconds has been reported for this test.^34^ The one study to investigate balance using the timed up-and-go test demonstrated no difference between groups for time to completion.^21^ Two studies reported findings for the five times sit-to-stand test.^20,29^ It is validated to measure the dynamic balance and functional mobility in older adults.^35^ A minimal clinical important difference of 2.3 seconds has been reported in the literature.^36^ One study demonstrated a significant difference between groups in favour of exercise.^29^ A meta-analysis was not able to be completed as appropriate data was not published and not provided after contact with the author.^20^

One study assessed balance via the semi-tandem and tandem stance balance tests.^20^ These tests are a measure of static balance. Data was not published or provided after author contact; therefore, no outcome was able to be analysed.

### Health outcomes

**Figure 4.**
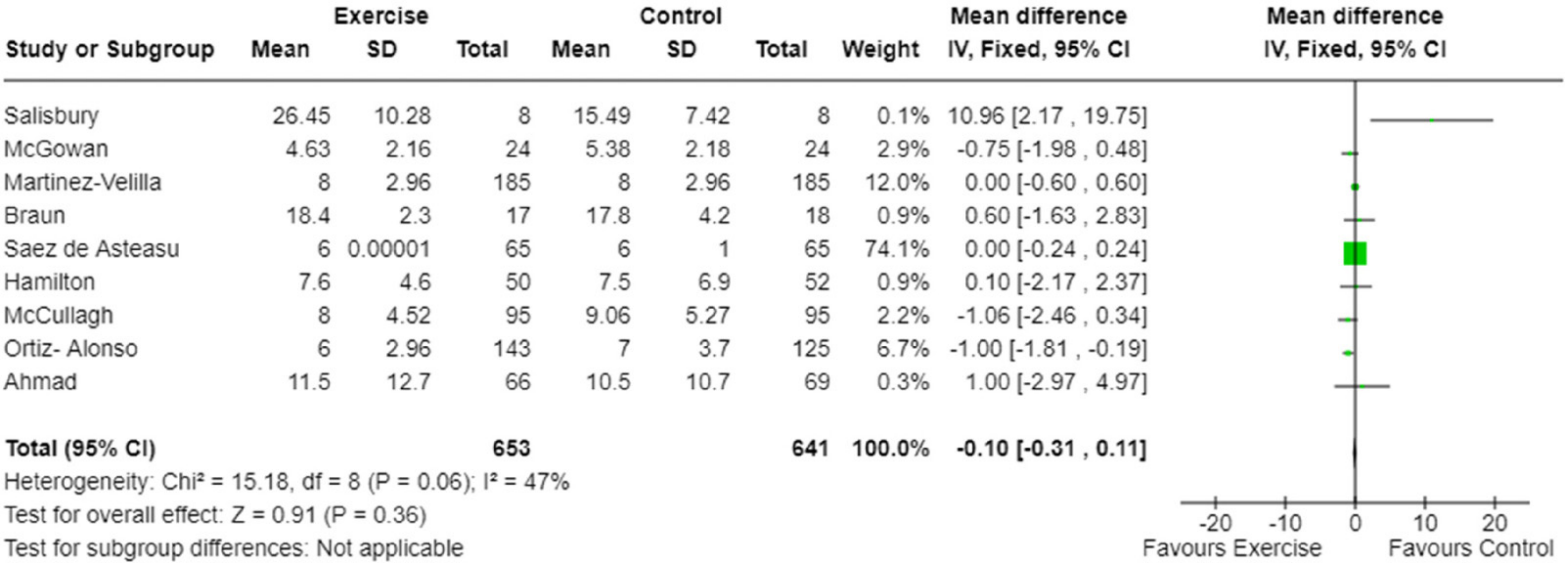
Meta-analysis of the effect of exercise intervention on hospital length of stay.

Length of hospital stay was reported in nine studies.^9,20–22,25–27,29,30^ Meta-analysis demonstrated no difference for total length of hospital stay between exercise intervention and usual care (Figure 4). The certainty of the evidence was moderate (Table 4) with moderate heterogeneity (I^2^ = 47%, Figure 4).

**Figure 5.**
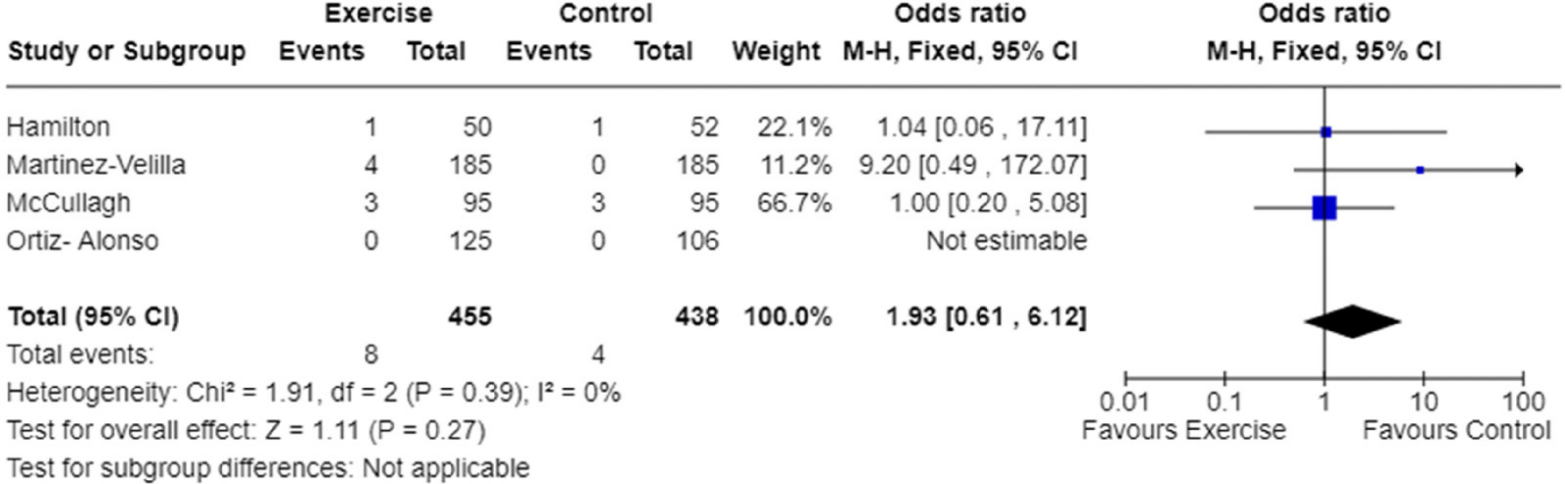
Meta-analysis of the effect of exercise intervention on in-hospital falls count.

In-hospital falls count was reported in four studies.^9,22,25,27^ Meta-analysis demonstrated the odds of falling while in hospital were not significantly different between the exercise and usual care groups (Figure 5). The quality of evidence was low (Table 4), and heterogeneity was low (I^2 ^= 0%, Figure 5).

Thirty-day readmissions were reported in three studies.^9,22,25^ Two studies reported no significant difference between groups,^9,23^ and one study reported a significant difference between groups in favour of the control group.^26^ Different data reporting methods (odds ratios, mean and standard deviation, median and interquartile range) for readmissions across the three studies meant meta-analysis could not be completed.

**Figure 6.**
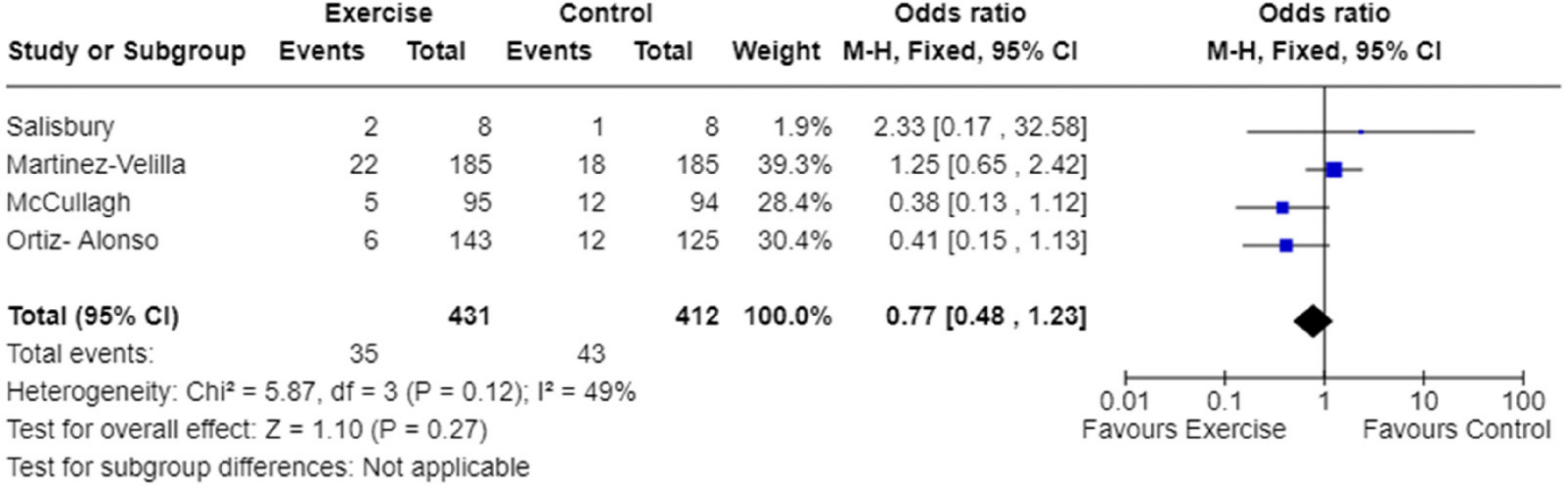
Meta-analysis of the effect of exercise intervention on mortality rate.

Three-month mortality rates were reported in four studies.^9,25,29,30^ Meta-analysis demonstrated the odds of mortality within three months of discharge were not significantly different between exercise intervention and usual care groups (Figure 6). The quality evidence was low (Table 4), and heterogeneity was moderate (I^2 ^= 49%, Figure 6).

## Discussion

This systematic review with meta-analyses pooled physical outcome and health outcome data to determine the effect of exercise interventions administered to patients on acute medical wards compared with usual care or no exercise intervention. The main findings of this review were significant differences between groups for the physical outcomes of aerobic capacity and maximum isometric strength in favour of exercise intervention. There were no significant differences for balance or any of the included health outcomes of length of stay, in-hospital falls count, readmission rates and three-month mortality. These findings need to be considered with caution due to the low certainty of evidence.

Improvements in aerobic capacity (determined by walking distance) and maximum isometric strength support previous findings that hospital-acquired functional decline can be mitigated by implementation of ward-based exercise.^6–10^ Improvements in walking ability have been linked with improved self-assessed overall health in older adults.^37^ To make improvements in aerobic capacity, it does not appear to matter if the exercise intervention included walking alone or was combined with resistance training. This aligns with findings from a previous review^38^ that reported improvements in aerobic capacity in hospital inpatients receiving an exercise intervention.

While the meta-analysis found a significant improvement in isometric muscle strength, this may not fully represent physical capacity as movement, by definition, is dynamic. To gain a full understanding of the benefits of these exercise interventions on physical capacity, it may be important to also include dynamic muscle strength measures.

No differences between exercise intervention and usual care were found for any of the health outcomes. Confounding factors such as number of complications or how unwell the sample group was may have influenced these findings. Another simple possibility for lack of difference is that participants may have been discharged from the hospital before the effect of the intervention had occurred. From a length of stay perspective, it might have been the research protocol that dictated the lack of response, as the studies included were randomised-controlled trials. By definition, this should have ensured that all participants were admitted and evaluated for the same time period, and while some studies did control the length of the intervention,^9,23,24,26,29^ others did not.^20–22,25,27,28,30^ It is challenging to control all potential variables that may influence these health outcomes, and therefore difficult to clearly define the effect of an on-ward exercise intervention on health outcomes.

The age range of the included participants for the current review was younger than previous systematic reviews. The inclusion criteria for the current review allowed for studies with participants 18 years of age or older, while previously published systematic reviews have only included older adults (60 years and older).^38^ However, even with the wide age range criteria for this review, only two studies included adults younger than 60 years of age,^23,28^ and the youngest mean age was 51 years.^23^ Older adults have an attenuated response to retraining after immobilisation compared with young adults,^39^ and a small decline in physical capacity in older adults has the potential to accelerate frailty, increasing the risk of adverse outcomes.^40^ There is, however, evidence that younger adults are also at risk of declining muscle function if insufficient physical activity is completed,^41^ creating opportunity to further research the effect of decreased physical capacity on post discharge health outcomes in adults aged under 60 years.

Other health measures such as quality of life, health service utilisation and participation in activity post discharge may provide more meaningful data on the impact of in-hospital exercise in this population. However, these measures are not commonly reported in studies investigating on-ward exercise interventions. This presents further opportunity for research to investigate the impact of on-ward exercise on other health outcomes.

There are limitations of this systematic review and meta-analyses that have the potential to influence the findings. The meta-analyses included a small number of studies that were of low to moderate quality, meaning the main findings need to be interpreted with caution. A search of the grey literature was not conducted, which may have meant data that has not been published in peer-reviewed journals were not identified.

In conclusion, exercise prescribed on the acute medical ward improves aerobic capacity and maximum isometric strength compared with usual care, indicating on-ward exercise prescription should focus on these physical outcomes. There was no effect of exercise intervention on the physical outcome of balance or the health outcomes of length of stay, in-hospital falls count, readmission rates or three-month mortality.

**Clinical messages**

- Exercise on a medical ward can improve aerobic capacity and muscle strength, which can potentially decrease frailty in this population.
- On-ward exercise interventions should focus on aerobic capacity and muscle strength rather than balance exercises.
- Further investigations are required to determine the most effective exercise prescription to modify disability associated with a hospital stay.

## Supplemental Material

sj-docx-1-cre-10.1177_02692155241240637 - Supplemental material for Effectiveness of exercise intervention on physical and health outcomes in patients admitted to an acute medical ward: A systematic review and meta-analysis Supplemental material, sj-docx-1-cre-10.1177_02692155241240637 for Effectiveness of exercise intervention on physical and health outcomes in patients admitted to an acute medical ward: A systematic review and meta-analysis by Jane L McCaig, Brett A Gordon and Carolyn J Taylor in Clinical Rehabilitation
